# Choriocarcinoma Syndrome: A Rare and Serious Complication of Testicular Germ Cell Tumors

**DOI:** 10.7759/cureus.18681

**Published:** 2021-10-11

**Authors:** Ziad Abuhelwa, Waleed Kassabo, Ying Ning, Majdal Hjouj, Abhijit Saste

**Affiliations:** 1 Internal Medicine, The University of Toledo, Toledo, USA; 2 Oncology, The University of Toledo, Toledo, USA; 3 Internal Medicine, Al-Quds University, Jerusalem, PSE; 4 Oncology, ProMedica Toledo Hospital, Toledo, USA

**Keywords:** chemotherapy, radiation, germ cell tumors, metastasis, choriocarcinoma

## Abstract

Choriocarcinoma syndrome is a rare complication of metastatic germ cell tumors. The hallmark of the condition is metastatic tumor hemorrhage. We describe the case of a 28-year-old man with metastatic testicular choriocarcinoma who presented with dyspnea on exertion and lightheadedness. Symptoms started two days after completing cycle one of fractionated bleomycin, etoposide, and cisplatin (BEP) chemotherapy. Laboratory investigations showed severe anemia, with a hemoglobin of 5.4 mg/dL (normal: 13-17 mg/dL). His baseline hemoglobin was 15.1 mg/dL before chemotherapy initiation. Coagulation and hemolysis workup showed no significant evidence of disseminated intravascular coagulopathy or autoimmune hemolysis. Imaging showed a significant increase in the size of previous metastatic liver lesions with surrounding hypodensity representing hemorrhage. He was admitted to the intensive care unit and started on massive transfusion protocol. On the same day, he developed a maroon-colored stool. Urgent upper endoscopy showed blood in the entire stomach and the second part of the duodenum spurting out through the ampulla, which suggested bleeding from metastatic liver lesions into the biliary tree. No defined culprit vessel was identified on visceral angiography. Endoscopic and surgical interventions were unlikely to be successful in controlling the bleeding due to the diffuse nature. He underwent one dose of radiation therapy to the abdomen, which was successful in controlling the bleeding. He survived, and his chemotherapy was switched to etoposide, ifosfamide, and cisplatin (VIP) with no further episodes of bleeding.

## Introduction

Testicular neoplasms are the most common solid malignancies in males between the ages of 15 and 34 years [1]. Testicular germ cell tumors (TGCT) constitute about 95% of testicular neoplasms, and they can be classified histologically into seminomatous and non-seminomatous TGCT [2]. Cisplatin-based chemotherapy secures a five-year survival rate of more than 95% in patients with TGCS [3]. Choriocarcinoma, the most aggressive and least common type, presents as an element of approximately 10 percent of testicular mixed GCTs [4]. It is usually associated with early widespread hematogenous metastasis and elevated serum human chorionic gonadotropin [5]. Choriocarcinoma syndrome (CCS) is a rare type of tumor lysis, manifesting as serious life-threatening hemorrhage at metastatic sites of advanced germ cell tumors containing large elements of choriocarcinoma. Unfortunately, there are no specific guidelines on the management and prevention of CCS, and immediate recognition of this condition is essential to initiate early treatment and improve the chance of recovery.

## Case presentation

A 28-year-old man presented to the emergency department due to dyspnea on exertion of one-day duration. He was recently discharged from the hospital after completion of bleomycin, etoposide, cisplatin (BEP) chemotherapy for a newly diagnosed metastatic testicular choriocarcinoma. Dyspnea started two days after discharge and it gradually worsened. He also endorsed associated left-sided chest pain. The chest pain was sharp in character and increased with breathing. He rated the severity of the pain as 6 out of 10 per the visual analog scale. He also complained of lightheadedness, dizziness, and blood-tinged sputum. A review of systems was otherwise negative for fever, chills, cough, nausea, vomiting, headache, numbness, weakness, hematuria, or hematochezia. He denied cigarette smoking, alcohol, or illicit drug use.

His past medical history is significant for a newly diagnosed metastatic testicular choriocarcinoma stage IIIC (cTX, cNX, pM1b, S3). At that time, he had multiple scattered metastatic hepatic, pulmonary, and retroperitoneal lesions. Additionally, he had a single small, 4 mm, metastatic, right occipital lobe lesion. He was admitted to the hospital and started on fractionated bleomycin, etoposide, cisplatin (BEP) chemotherapy. He tolerated the first BEP cycle very well and he was discharged home in a good condition.

Physical examination showed an acutely ill and pale patient. Cardiovascular examination revealed tachycardia with regular rate and rhythm. Lung auscultation was unremarkable. Abdominal examination showed mild tenderness over the upper abdomen with no rebound or rigidity. Neurological examination did not reveal any focal neurological deficit.

Laboratory investigations showed hemoglobin of 8.5 mg/dL (normal: 13-17 g/dL). Baseline hemoglobin level before chemotherapy initiation was 15.1 mg/dL. Serum chemistry showed blood urea nitrogen 21 mg/dL (normal: 5-23 mg/dL), creatinine 0.61 mg/dl (normal: 0.60-1.30 mg/dL), potassium 4.2 mg/dL (normal 3.5-5 mg/dL), uric acid 3.7 mg/dL (normal: 3.4-7.0 mg/dL), aspartate transaminase 21 U/L (normal: 0-41 U/L), and alanine transaminase 38 U/L (normal: 0-40 U/L). Serum beta-human chorionic gonadotropin was 199,579 IU/L. Coagulation studies showed prothrombin time 16 sec (normal: 9.5-12.6 sec), international normalized ratio 1.4 (normal: 0.9-1.2), partial prothrombin time 26 sec (normal: 26-37 sec), D-dimer level was 6720 ng/ml (normal: <255 ng/mL DDU), and fibrinogen 230 mg/dL (normal: 190-480 mg/dL). Hemolysis workup revealed haptoglobin <30 (normal: 32-228 mg/dL), lactate dehydrogenase 585 U/L (normal: 100-235 U/L), total bilirubin 1.1 mg/dL (normal: 0.3-1.2 mg/dL), and negative direct Coombs test. Peripheral blood showed anemia and few schistocytes suggesting mild hemolysis.

**Figure 1:**
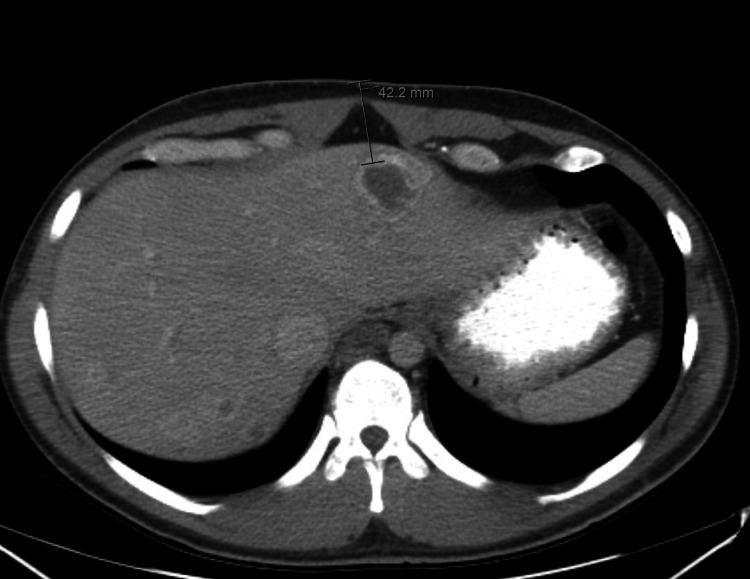
CT of the abdomen showing hepatic metastatic lesions at the time of presentation and prior to chemotherapy initiation

**Figure 2:**
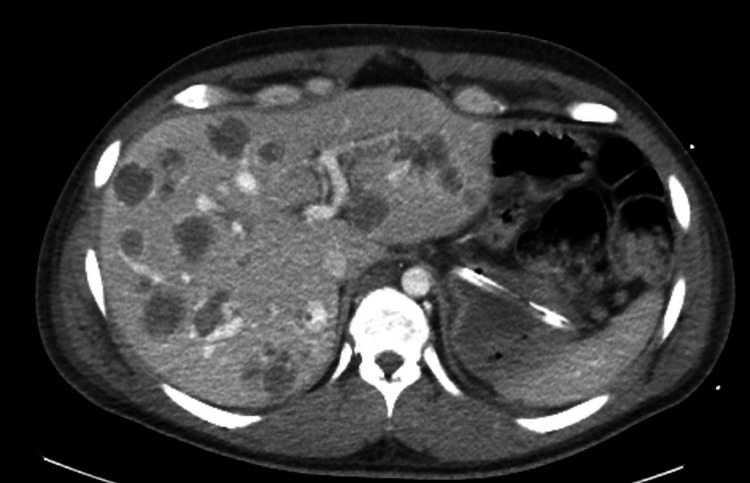
CT of the abdomen showing a significant increase in the size of all hepatic metastatic lesions, with lesions appearing more hypodense, representing hemorrhage

Chest computed tomography (CT) angiography did not show any evidence of pulmonary embolism. Imaging CT of the abdomen and pelvis showed a significant increase in the size of all hepatic metastatic lesions with more surrounding hypodensity compared to the time of initial diagnosis (Figures 1-2). Brain CT showed increased surrounding vasogenic edema of the right occipital lobe metastatic lesion.

He was transferred to the intensive care unit for close monitoring. His hemoglobin was trending down despite transfusion with no apparent signs of external bleeding. He received tranexamic acid and was started on the massive transfusion protocol. Later, he developed severe melanotic stool with bright red blood streaks. Repeated hemoglobin was 5.4 mg/dL. He was intubated for airway protection and mechanical ventilation was initiated. Urgent upper endoscopy showed blood in the entire examined stomach and duodenum, blood was spurting out through the ampulla of Vater, which was suggestive of bleeding from the biliary tree. He underwent visceral angiography, which demonstrated diffuse blush throughout the entire liver with no defined culprit vessel. Due to the diffuse nature of his visceral bleeding, endoscopic and surgical interventions were unlikely to be successful in controlling the bleeding. In addition to continued supportive treatment, he received one dose of radiation therapy to the abdomen, which helped in controlling his diffuse bleeding. Over the course of the next five days, his clinical condition stabilized and he was discharged home. His chemotherapy regime was switched from BEP to etoposide, ifosfamide, cisplatin (VIP) with no further episodes of bleeding.

## Discussion

CCS is a life-threatening complication associated with TGCT. It has been only reported a handful of times. Metastatic choriocarcinoma is very vascular and tends to progress to life-threatening hemorrhage at the sites of metastasis [6]. Diffuse alveolar hemorrhage secondary to pulmonary metastasis is the most common manifestation. Hemorrhage in other organs, including the liver, kidneys, bones, small intestine, and brain, was also reported [7]. CCS is not a well-known entity due to its extreme rarity. In 1984, the first case of CCS was reported by Logothetis CJ in Japan [8-9]. CCS might develop during the course of treatment, or it may be the presenting symptom of advanced-stage disease burden [10]. Zeitjian et al. reported a case of a male patient with metastatic choriocarcinoma who developed melena and hemoptysis before starting chemotherapy. He was started urgently on VIP chemotherapy but, unfortunately, he developed acute respiratory distress syndrome, and in spite of maximal respiratory support, he passed away [9]. Kandori et al. reported a case of pulmonary metastatic choriocarcinoma who developed pulmonary hemorrhage after chemotherapy [8].

We present a case of a young man with metastatic testicular choriocarcinoma who presented with severe anemia due to bleeding from metastatic lesions. The bleeding was mainly from the hepatic metastatic lesions. Additionally, bleeding around the brain metastatic lesion was noted; however, it was not clinically significant. Symptoms started around two days after he received the first cycle of fractioned BEP chemotherapy. It was not amenable to any endoscopic or surgical interventions and was controlled with one dose of radiation therapy to the abdomen.

Unfortunately, there are no specific guidelines to prevent or manage CCS. It is unclear whether chemotherapy contributes to or hastens the hemorrhage. Some of the reported cases developed CCS before starting chemotherapy while others after chemotherapy. The management of CCS revolves around early recognition, a multidisciplinary approach, and organ-specific interventions to control the hemorrhage. This is evident in a case of pulmonary hemorrhage that was successfully managed with lobectomy and prompt surgical intervention [11]. Moreover, Komori et al. reported a case with metastatic extragonadal germ cell tumor who presented with hemoperitoneum that was treated surgically [12]. In our case, there were no well-defined culprit vessels to embolize or treat surgically. Radiation therapy to the abdomen was successful in controlling the massive hemorrhage in our case. Our patient survived, and he was started on VIP chemotherapy with no further episodes of bleeding.

## Conclusions

CCS is a rare and life-threatening complication characterized by bleeding from germ-cell tumor metastatic sites. A high index of suspicion and early recognition are needed. CCS should be suspected when a patient is presenting with symptoms and signs of bleeding in addition to radiographic evidence of worsening metastatic lesions. There are no specific prevention and management guidelines. Radiation therapy to sites of bleeding can be helpful in controlling the bleeding.
